# CAR T-Cells for CNS Lymphoma: Driving into New Terrain?

**DOI:** 10.3390/cancers13102503

**Published:** 2021-05-20

**Authors:** Philipp Karschnia, Jens Blobner, Nico Teske, Florian Schöberl, Esther Fitzinger, Martin Dreyling, Joerg-Christian Tonn, Niklas Thon, Marion Subklewe, Louisa von Baumgarten

**Affiliations:** 1 Department of Neurosurgery, Division of Neuro-Oncology, Ludwig Maximilians University School of Medicine, Marchioninistrasse, 1581377 Munich, Germany; jens.blobner@med.uni-muenchen.de (J.B.); nico.teske@med.uni-muenchen.de (N.T.); esther.fitzinger@web.de (E.F.); Joerg.Christian.Tonn@med.uni-muenchen.de (J.-C.T.); Niklas.Thon@med.uni-muenchen.de (N.T.); 2 German Cancer Consortium (DKTK), Partner Site Munich, 80336 Munich, Germany; Marion.Subklewe@med.uni-muenchen.de; 3 Department of Neurology, Ludwig-Maximilians-University School of Medicine, 81377 Munich, Germany; Florian.Schoeberl@med.uni-muenchen.de; 4 Department of Medicine, Hematology & Oncology Division and Cellular Immunotherapy Program, Ludwig-Maximilians-University School of Medicine, 81377 Munich, Germany; Martin.Dreyling@med.uni-muenchen.de; 5 Gene Center of the LMU Munich, Laboratory for Translational Cancer Immunology, 81377 Munich, Germany

**Keywords:** refractory, relapsed, central nervous system, adoptive, survival, immunotherapy

## Abstract

**Simple Summary:**

Primary or secondary central nervous system (CNS) lymphoma is frequently associated with a poor prognosis. CAR T-cells are being established as a relevant treatment approach in hematological B-cell malignancies. Unfortunately, most clinical studies on chimeric antigen-receptor (CAR) T-cells have excluded patients with CNS involvement but several clinical trials on CAR T-cell therapy in CNS lymphoma patients are currently ongoing. Preclinical and preliminary clinical data suggest an overall acceptable safety profile and considerable anti-tumor effects might be extrapolated for CAR T-cell therapy in CNS lymphoma.

**Abstract:**

Primary CNS lymphomas (PCNSL) represent a group of extranodal non-Hodgkin lymphomas and secondary CNS lymphomas refer to secondary involvement of the neuroaxis by systemic disease. CNS lymphomas are associated with limited prognosis even after aggressive multimodal therapy. Chimeric antigen receptor (CAR) T-cells have proven as a promising therapeutic avenue in hematological B-cell malignancies including diffuse large B-cell lymphoma, B-cell acute lymphoblastic leukemia, and mantle-cell lymphoma. CARs endow an autologous T-cell population with MHC-unrestricted effectivity against tumor target antigens such as the pan B-cell marker CD19. In PCNSL, compelling and long-lasting anti-tumor effects of such therapy have been shown in murine immunocompromised models. In clinical studies on CAR T-cells for CNS lymphoma, only limited data are available and often include both patients with PCNSL but also patients with secondary CNS lymphoma. Several clinical trials on CAR T-cell therapy for primary and secondary CNS lymphoma are currently ongoing. Extrapolated from the available preliminary data, an overall acceptable safety profile with considerable anti-tumor effects might be expected. Whether these beneficial anti-tumor effects are as long-lasting as in animal models is currently in doubt; and the immunosuppressive tumor microenvironment of the brain may be among the most pivotal factors limiting efficacy of CAR T-cell therapy in CNS lymphoma. Based on an increasing understanding of CAR T-cell interactions with the tumor cells as well as the cerebral tissue, modifications of CAR design or the combination of CAR T-cell therapy with other therapeutic approaches may aid to release the full therapeutic efficiency of CAR T-cells. CAR T-cells may therefore emerge as a novel treatment strategy in primary and secondary CNS lymphoma.

## 1. Introduction

Primary central nervous system lymphoma (PCNSL) represent a rare group of extranodal B-cell non-Hodgkin lymphomas arising from the brain parenchyma, spinal cord, eyes, or meninges without systemic, extra-axial involvement [1]. Such tumors account for 2% of all primary central brain tumors [2,3]. Antimetabolites including methotrexate and cytarabine represent the backbone of anti-PCNSL therapy, and may be followed by consolidation radiotherapy or high dose chemotherapy and autologous stem cell transplantation (Supplementary Figure S1) [4]. The addition of chemotherapy to the former standalone radiotherapy has translated into substantially improved survival [5]; however, PCNSL is still associated with limited outcome compared to extra-axial disease and a median survival of less than three years [6]. Importantly, radiotherapy is frequently accompanied by disabling neurotoxicity including decline in cognitive function, and such effects need to be carefully weighed against potential benefits in terms of survival [7]. Secondary CNS lymphomas refer to secondary involvement of the neuroaxis by systemic disease, and often indicate aggressive disease with unfavorable survival compared to systemic disease only [8]. Median survival after diagnosis of secondary CNS lymphoma is only about four months [8]. The identification of new therapeutic approaches for primary and secondary CNS lymphomas is therefore urgently warranted.

Adoptive immunotherapy with chimeric antigen receptor (CAR) T-cells has emerged as an efficient therapy for relapsed or refractory hematological malignancies [9]. Following viral transduction, CARs direct the killing properties of an autologous T-cell population against a tumor cell antigen. To increase persistence, activity, and expansion, CARs are equipped with a costimulatory domain [10]. Numerous clinical studies demonstrated substantial response rates for CAR T-cells directed against the pan-B cell antigen CD19 in patients with diffuse large B-cell lymphoma [11], B-cell acute lymphoblastic leukemia [12], and mantle-cell lymphoma [13]. Five CAR T-cell products are currently available for commercial use in the United States and the European Union and constitute a major breakthrough in the treatment of hematological cancers. Given that almost all CNS lymphoma manifestations express CD19 [14,15], there is a strong biologic rationale to treat such patients with CD19-directed CAR T-cells. However, there is no definitive conclusion on whether this indeed represents a promising therapeutic avenue. We herein provided a review on the available literature for CAR T-cells in the treatment of PCNSL and also secondary CNS lymphoma. We summarized recent preclinical and clinical data on CAR T-cell therapy for primary and secondary CNS lymphoma, discussed challenges when treating primary brain tumors with CAR T-cells, and hypothesized on future directions of the field.

## 2. Preclinical and Clinical Data

### 2.1. Preclinical Data

Anti-tumor effects of CD19-directed CAR T-cells against PCNSL have not only been demonstrated in vitro, but also in murine in vivo models [16,17]. Mulazzani et al. designed an orthotopic PCNSL model by combining a chronic cranial window with two-photon intravital microscopy, allowing the repetitive visualization of brain tumor growth [16]. A single dose of intracerebrally injected CD19-directed CAR T-cells was not only able to mediate regression, but also to completely eliminate established PCNSL in two out of three animals. These substantial anti-tumor effects lasted up to half a year until experiments were terminated, and CAR T-cells resided in the brain parenchyma as well as in draining and non-draining lymph nodes throughout the observation period. Importantly, intravenous CAR T-cell injection was associated with a low number of tumor-infiltrating CAR T-cells and therefore not able to sufficiently control PCNSL growth. Although the authors speculated that this might be due to poor trafficking of CAR T-cells across the blood–brain barrier or a rather low number of intravenously injected cells, the final mechanisms behind this observation were not elucidated. Importantly, the study was limited by the use of immunoincompetent mice lacking functional T-cells (but retain B-cells) as human lymphoma cells were utilized.

PCNSL regression after local but not intravenous administration of CAR T-cells was recently corroborated in another immunoincompetent mouse model (lacking function T- and B-cells) of PCNSL [18]. Wang et al. induced orthotopic PCNSL growth by intracranial injection of human lymphoma cell lines. CD19-directed CAR T-cells were either delivered via a single intraventricular or intravenous infusion. Bioluminescence was measured to quantify tumor growth in vivo over the course of weeks, and only intraventricularly injected CAR T-cells were able to control PCNSL growth. Single-cell RNA analysis of CAR T-cells sampled from the bone marrow of post-treatment mice, in vitro culture of CAR T-cells in either cerebrospinal fluid (CSF) or medium, and further mechanistic analyses showed that exposure to the CSF results in a distinct anti-tumor and memory effectivity of CAR T-cells.

These findings made from preclinical studies appear therefore promising in controlling PCNSL; however, they have not yet been validated in immunocompetent animal models. Given that only a limited number of preclinical studies on CAR T-cells and PCNSL is available, a high level of suspicion is therefore required when interpreting these results; however, some anti-tumor effects against PCNSL and CNS lymphoma in general might be assumed.

### 2.2. Clinical Data

Patients with active involvement of the brain were excluded from almost all clinical trials on CAR T-cells, mainly due to dreaded more severe neurotoxic side effects. These trials have resulted in US Food and Drug Administration (FDA) approval of CD19-directed CAR T-cells for patients with systemic but not CNS disease [11,12]. So far, only three studies analyzing the clinical efficiency of CAR T-cells in patients with primary or secondary CNS lymphoma are available to date, all of them using CAR T-cells targeting CD19 [19,20,21] (Table 1).

In 2017, a first case report on CAR T-cell efficacy in secondary CNS lymphoma was published [19]. Abramson et al. enrolled a 68-year-old female with refractory diffuse large B-cell lymphoma in the TRANSCEND-NHL-001 trial on the CAR T-cell product lisocabtagene maraleucel (formerly known as JCAR017). After T-cell apheresis and prior to lymphodepletion and CAR T-cell infusion, re-staging studies were provided and a new right temporal mass consistent with disease involvement of the CNS was noted on imaging. The patient proceeded with lymphodepletion and intravenous CAR T-cell infusion (NCT02631044) as initially planned, and complete remission of the cerebral lymphoma site was seen one month after infusion. Of note, this remission was durable and ongoing for 12 months at the time the report was published. Neither cytokine release syndrome nor neurotoxicity was noted.

Another CD19-directed CAR T-cell product, tisagenlecleucel (formerly known as CTL019), has been approved in 2017 for large B-cell lymphoma patients with systemic but also secondary (not primary) CNS involvement. Based on the granted FDA approval, Frigault et al. treated and reported on a retrospective cohort of eight patients with secondary CNS involvement of the brain, spine, and leptomeninges [20]. All patients received lymphodepletion and a single intravenous CAR T-cell infusion of tisagenlecleucel (0.6 × 10^8^ to 6.0 × 10^8^ CAR T-cells). Only mild neurotoxic or systemic side effects were encountered, and none of these patients experienced CAR T-cell-mediated toxicities necessitating therapy with the anti-interleukin 6-receptor antagonist tocilizumab or steroids. Response assessment on day 28 after CAR T-cell infusion showed complete response in two patients, partial response in two more patients, and disease progression in four patients (including two fatalities due to progressive disease). Further follow-up on day 90 revealed ongoing disease control in three of the four patients who initially responded to CAR T-cells, and long-term follow up on day 180 was available in one of those patients showing complete response.

These results suggesting considerable anti-tumor effects in the treatment of CNS disease were recently corroborated by preliminary data from an ongoing prospective trial of CD19-directed CAR T-cells for B-cell non-Hodgkin lymphoma (NCT02153580) [21]. The studied CAR T-cell product is modified to express a truncated human epidermal growth factor receptor, which may serve as an antibody target to rapidly eliminate CAR T-cells in vivo in case of severe toxicities. Three patients with primary and four patients with secondary CNS lymphoma were treated by intravenous CAR T-cell infusion (2 × 10^8^ to 6 × 10^8^ CAR T-cells) following lymphodepletion, whereas when no life-threatening toxicities occurred, tocilizumab was provided for moderate cytokine release syndrome in two patients and steroids for neurotoxicity in three patients. Four patients had disease responses to CAR T-cells with one patient showing complete response and three patients showing partial response. On a cautionary note, follow-up time was only in the range of several weeks and it is unclear whether this response was durable.

Data from longer follow-up intervals after treatment of CNS disease were reported from Li et al. (ChiCTR-OPN-16008526) [22]. One patient with primary and four patients with secondary CNS lymphoma each received one intravenous infusion of CD19-directed (2.2 × 10^6^ to 7.1 × 10^6^/kg body weight) and one infusion of CD22-directed CAR T-cells (3.1 × 10^6^ to 7.0 × 10^6^/kg). CD22 is another pan B-cell marker which offers an additional target in the case of CD19 antigen loss [24]. In this cohort, one case of mild neurotoxic symptoms and one case of high-grade neurotoxicity was encountered which necessitated the use of steroids and plasmapheresis. All 5 patients responded within 60 days after CAR T-cell administration including two complete responses. However, four patients relapsed within three to eight months, and median progression-free survival was three months. Despite tumor relapse, tumor tissue analysis and CSF studies in one patient showed persistent target antigen expression and detectable CAR T-cells. The authors speculated that the immunosuppressive tumor microenvironment providing resistance against CAR T-cells might have contributed to tumor recurrence. However, the authors lacked sufficient evidence for this theory.

Based on the encouraging results of above-mentioned studies, different clinical phase I and phase II trials are currently testing safety and efficiency of CD19 CAR T-cells in primary and secondary CNS lymphoma patients (Table 2).

## 3. Challenges for CAR T-Cells in CNS Lymphoma

The above-mentioned studies suggest an acceptable safety profile of CAR T-cells for CNS lymphoma. Furthermore, considerable anti-tumor effects have been reported. Whether these anti-tumor effects are as long-lasting and profound as it has been described for extracranial disease might be in doubt. A number of CNS-specific aspects may hamper clinical success of such therapies.

### 3.1. Immune-Escaping Tumor Properties

Primary brain tumors including PCNSL represent complex compositions of neoplastic and non-neoplastic cells which individually contribute to tumor formation [25]. Tumor-associated macrophages and microglia (TAM/M) constitute the majority of non-neoplastic cells in PCNSL, and these cells create a particular immunosuppressive pre-metastatic niche which facilitates tumor cell extravasation, survival, and expansion [26]. A spectrum of TAM/M activation phenotypes have been defined between the pro-inflammatory, anti-tumor M1 and the anti-inflammatory, pro-tumor M2 phenotype [27]. Accordingly, higher numbers TAM/M polarized towards M2 phenotype are associated with less favorable outcome in PCNSL [28]. In addition, immunosuppressive cytokines are strongly expressed in PCNSL, whereas cytokines promoting cell-based immune response are downregulated [29]. To mitigate the immunosuppressive milieu, different approaches including CAR T-cells expressing inducible proinflammatory cytokines [30] or combination therapies with checkpoint inhibitors [31] are currently being investigated in preclinical trials.

### 3.2. Role of the Blood–Brain Barrier and Route of CAR T-Cell Application

In addition to metabolic barriers for tumor infiltration by CAR T-cells, physical barriers including the blood–brain barrier may limit treatment success. Under physiologic conditions, the brain is virtually free of leucocytes, and their influx is tightly regulated. CAR T-cells have been shown to migrate across the blood–brain barrier and can be found in brain and CSF [16,32]. In turn, locally injected CAR T-cells have not only been found to travel to distant sites within the CSF but can also be detected in the systemic circulation [16,33]. After intravenous injection, the number of CAR T-cells within the CSF seems generally lower than in the systemic circulation [32]. In preclinical models, intravenous administration of CAR T-cells for CNS lymphoma but also other brain tumors such as glioblastoma has provided considerable anti-tumor effects [32,34]. However, direct comparison of different routes of application indicate that local delivery may be associated with improved treatment response in brain tumors [16,35]. Importantly, immunodeficient PCNSL mouse models may lack proper function of circulating B-cells and CD19-directed CAR T-cells may therefore not encounter their target immediately after intravenous injection (in contrast to local delivery). This may impair CAR T-cell expansion, and thus underestimate their anti-tumor effectivity. However, insufficient anti-tumor effects after intravenous injection of CAR T-cells have not only been observed in the murine model by Wang et al. [18] who used NOD scid gamma mice (lacking B- and T-cell function), but also in the murine model by Mulazzani et al. [16] who made use of Foxn1^nu/nu^ mice (lacking T- but not B-cell function). However, the murine CD19 on B-cells from Foxn1^nu/nu^ mice differs substantially from human CD19 which the CAR T-cells were directed against in the study by Mulazzani et al. It might therefore be indeed speculated that these models may underestimate CAR T-cell effectivity.

Clinical studies for glioblastoma show that local CAR T-cell delivery (delivery into a resection cavity or administration into the CSF) is feasible and effective [16,18,35]. Several clinical studies using local delivery of CAR T-cells to treat primary brain tumors other than PCNSL are currently recruiting (NCT03500991, NCT03638167, NCT04185038). Local injection of CAR T-cells for CNS lymphoma has so far not yet been conducted; however, it might represent an approach warranting evaluation especially for patients with primary CNS lymphoma given the exclusive CNS involvement.

### 3.3. Antigen Loss

Another factor contributing to recurrence after CAR T-cell therapy may be the loss or downregulation of the tumoral target antigens. Of patients with B-cell leukemia, 7–25% experience CD19-negative relapse [12,36]. The frequency of antigen loss in lymphoma is less clear given that biopsies are rarely obtained during relapse. However, several of such cases have been described after CAR T-cell therapy for systemic lymphoma [37], and antigen loss might therefore be also relevant for PCNSL [38]. In the future, (re-)biopsies of cerebral manifestations should be encouraged for antigenic profiling of the new lesion in order to substantiate the presence of druggable targets. Approaches to prevent or circumvent antigen loss as potential escape mechanism will also need to be evaluated in CNS lymphoma.

### 3.4. Adverse Effects of CAR T-Cells

CAR T-cell therapy directed against CD19, but also other antigens might be accompanied by unique toxicities including cytokine release syndrome (CRS), immune effector cell-associated neurotoxicity syndrome (ICANS), on-target–off-tumor toxicities, and prolonged cytopenia [39,40].

CRS represents the most commonly encountered adverse effect and is characterized by a systemic increase of pro-inflammatory cytokines translating into sepsis-like symptoms [41]. A high number of up to 93% of patients with extra-axial lymphoma treated with CAR T-cells may experience some degree of CRS, and one out of ten patients may experience severe symptoms necessitating treatment at an intensive care unit [39]. So far, in the treatment of CNS lymphoma, only mild cases of CRS were seen with a frequency similar to what has been observed for systemic disease [11,12,21].

ICANS is the second most commonly observed toxicity following CAR T-cell therapy. Clinical presentation varies and includes moderate symptoms such as headaches, fatigue, or aphasia [42], but also more severe and potentially life-threatening symptoms such as seizures/status epilepticus, cerebral edema, and death [43]. Pathophysiology is likely multifactorial and involves IL-1- and IL-6-mediated systemic inflammation, blood–brain barrier disruption, endothelial activation, and cross-reactivity of CAR T-cells against brain tissue [44,45]. On-target–off-tumor toxicity refers to effects caused by CAR T-cells against non-pathogenic tissue due to shared expression of target antigens on neoplastic and healthy tissue. Parker et al. recently demonstrated by single-cell RNA sequencing and autopsy studies that brain mural cells, which are critical for blood–brain barrier integrity, express CD19 [45]. Thus, an on-target mechanism may contribute to the development of ICANS. The occurrence of severe ICANS has been associated with decreased survival after CAR T-cell therapy [46]. Treatment consists in the application of steroids. Tocilizumab, which can be used to treat CRS, does not seem to improve ICANS. As therapy escalation plasmapheresis, the application of immunoglobulins, and the IL1-antagonist anakinra may show beneficial effects in individual cases [47,48,49]. Although there has been major concern that treatment of CNS disease may be paralleled by strong neurotoxic symptoms, only one case of severe ICANS in a CNS lymphoma patient receiving CD19 and CD22 directed CAR T-cells has so far been described [22]. In clinical trials investigating CAR T-cell therapy for other primary brain malignancies like glioblastoma, only few cases with severe CRS and no clinical manifestation of ICANS were reported [50]. No data exist for locally injected CAR T-cells in CNS lymphoma therapy which would also circumvent direct exposition of brain mural cells to CAR T-cells, and future clinical trials will need to closely monitor for ICANS. In addition, long-term effects of CAR T-cell treatment on cognitive performance have been described [51], and the heavy pre-treatment burden including whole-brain radiotherapy of CNS lymphoma patients may aggravate such effects [7].

Hematological toxicities are among the most common, yet underreported adverse effects of CAR T-cell therapy [40]. As all commercially available CAR T-cell products are exclusively approved for patients with refractory or relapsed malignancies, all patients underwent extensive treatment prior to CAR T-cell infusion which frequently results in profound and long-lasting cytopenia. Moreover, lymphodepletion is usually used prior to CAR T-cell infusion as it may enhance anti-tumor efficacy by decreasing regulatory T-cells and myeloid-derived suppressor cells, increasing levels of proinflammatory cytokines, and enhancing innate immunity [52]. Such an approach is also often associated with profound cytopenia; however, the extent and frequency of observed cytopenia cannot fully be explained by lymphodepletion or prior chemotherapies alone and likely also includes CAR T-cell-mediated mechanisms [53]. A high level of suspicion is required as CAR T-cell patients are therefore highly susceptible for viral, bacterial, or fungal infections [54]. Problematically, therapeutic agents which alleviate cytopenia such as GM-CSF could potentially worsen other toxicities such as ICANS [55]. One could argue that local delivery of CAR T-cells (e.g., intratumoral injection of CAR T-cells or intraventricular application via indwelling ventricle catheter) holds the potential to decrease systemic adverse effects like cytopenia by decreasing circulating CAR T-cells in the blood stream; however, data on that subject remain scarce and future clinical trials will have to address different routes of administration for PCNSL immunotherapy.

### 3.5. Hematological Limitations: Lymphopenia and Autoimmune Diseases

Whereas patients with glioblastoma and primary brain tumors other than PCNSL often experience lymphopenia before, during, and after CAR T-cell therapy as stated above, patients with CNS lymphoma are at a particularly high risk due to aggressive myeloablative first-line therapies which also include the use of stem cell transplantation [56,57,58]. In selected patients with low lymphocyte counts, apheresis of an adequate quantity of autologous T-cells for CAR T-cell manufacturing might be challenging. Fortunately, with a continuous improvement in lymphapheresis protocols, sufficient yields of lymphocytes might be expected in most patients [59]. Of note, CAR T-cells from aged donors are of impaired quality including shorter persistence and less memory-like phenotypes and one may speculate that there might also be an association with more extensive pre-treatment burden [60].

Moreover, autoimmune disorders requiring immunosuppressive medication are overrepresented among patients with CNS lymphoma [61]. The pathomechanistic implications of this observations are not fully understood but likely involve Epstein–Barr virus-induced mutations and such patients might be at particular risk for less favorable outcome [62,63]. Importantly, individuals with autoimmune disease were excluded from the landmark clinical trials that resulted in the approval of commercial CAR T-cell products, and there is only little evidence whether CAR T-cell therapy may aggravate symptoms or not [64]. It remains to be shown whether CAR T-cell therapy is safe and beneficial also in patients with CNS lymphoma and a history of autoimmune disease.

## 4. Discussion: Future Perspectives

Given the substantial adverse effects of CAR T-cells, anti-tumor effects will need to be carefully weighed against such side effects. Improvement of CAR T-cell efficacy might therefore be critical to consider such a therapy in patients in which other therapeutic approaches might still be available.

### 4.1. CAR T-Cell Design

**Figure 1:**
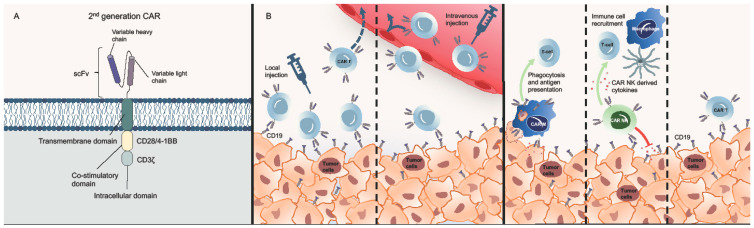
CAR design, route of CAR T-cell administration, and CAR cells. (**A**) Example of a second-generation CAR T-cell design used in the published clinical trials, incorporating a single-chain variable fragment (scFv) as an extracellular ligand recognition domain providing tumor antigen specificity, a transmembrane domain, an intracellular T-cell activating domain that includes a CD3 zeta chain (CD3ζ), and a co-stimulatory domain (CD28/4-1BB), included into the manufacturing process to ensure a more persistent CAR T-cell activity. (**B**) Only intravenous injection of CAR T-cells for lymphoma has been conducted so far. The blood–brain barrier may hamper delivery of CAR T-cells to the tumor environment whereas locally delivered CAR T-cells can travel to distant sites within the cerebrospinal fluid but can also be found in the systemic circulation. (**C**) CAR T-cells, natural killer cells, and macrophages all induce antigen targeted tumor cell elimination. CAR NK-cells recruit additional immune cells such as macrophages, T-cells, and dendritic cells, therefore improving anti-tumor activity. CAR-macrophages express proinflammatory cytokines and demonstrate antigen-specific phagocytosis, thereby presenting tumor antigens to regular T-cells. Abbreviations: CAR—chimeric antigen receptor; CAR NK—CAR natural killer cells; CAR T—CAR T-cells; CAR M—CAR-macrophages; DC—dendritic cell; scFv—single-chain variable fragment.

Recent CAR T-cell constructs have been evolving with novel design approaches emerging to optimize clinical efficacy and safety. The basic structure of every CAR consists of an extracellular ligand recognition domain, typically a single-chain variable fragment, providing tumor antigen specificity, a transmembrane domain, and an intracellular T-cell-activating domain that includes a CD3 zeta chain. Such ‘first-generation CARs’ showed limited efficacy in early clinical trials due to their insufficient signaling capability and low persistence [65,66]. To ensure a more sustained T-cell response, ‘second-generation CARs’ were modified to endow co-stimulatory domains such as CD28, 4-1BB, or OX40 [67,68]. Of interest, ‘second-generation CARs’ are the most widely used CAR T-cell constructs including all four commercially available CD19-directed CAR T-cell products (Figure 1A) [11,12,13]. Since a combination of multiple co-stimulatory domains could potentially enhance anti-tumor effects, ‘third-generation CARs’ incorporating both CD28 and 4-1BB co-stimulatory domains are currently being investigated in preclinical and clinical trials [69,70,71]. Such modifications may therefore increase expansion and activation of CAR T-cells which might be of particular importance in CNS lymphoma given its peculiar immunosuppressive properties [72].

In an effort to mitigate the effects of the immunosuppressive lymphoma microenvironment, featuring TAM/M activation and immunosuppressive cytokines, CARs expressing an additional transgenic inducible-cytokine have been designed [30]. Such ‘fourth generation CARs’ are also denoted as ‘T-cells redirected for antigen-unrestricted cytokine-initiated killing’ (TRUCKs) and combine the cell-mediated attack with the immune modulating properties of a transgenic cytokine, released upon tumor–antigen binding [73]. Through CAR-induced release, the cytokine is delivered in the tumor microenvironment inducing a pro-inflammatory (and anti-tumorous) micromilieu while alleviating systemic effects. One in vivo study using CEA-directed CAR T-cells with inducible IL-12 in a mouse model of CEA-positive tumors could even show elimination of antigen-negative tumor cells which could prove critical in relapsed or refractory malignancies demonstrating tumoral target antigen loss or downregulation [30].

To circumvent tumor recurrence due to loss of the target antigen, CAR T-cell products able to recognize multiple tumor antigens have been designed. As such, Tu et al. described a single PCNSL patient who was treated simultaneously with CD19- and CD70-directed CAR T-cells, and durable complete remission at 17 months follow-up was reported [74]. Moreover, the above-mentioned study from Li et al. used a combination of CD19- and CD22-directed CAR T-cells, but a less favorable outcome was seen in the reported cohort of five patients [22]. Nevertheless, tissue analysis at tumor recurrent suggested that antigen escape might have not contributed to recurrence.

Apart from improving CAR constructs, there is a growing interest in developing alternative cell lines endowing antigen-specificity such as CAR NK-cells and CAR-macrophages, both showing properties that could enhance anti-tumor activity (Figure 1B,C). In addition to CAR-targeted tumor cell elimination, CAR NK-cells exhibit natural cytotoxic activity against tumor cells independent of tumor antigen presentation [75]. Moreover, CAR NK-cells activate other immune cells such as macrophages, T-cells, and dendritic cells and are less likely to cause cytokine release syndrome or neurotoxicity [76]. In other primary CNS malignancies like glioblastoma, CAR NK-cells have already shown promising results in preclinical studies [77], and HER2-directed CAR NK-cells for glioblastoma are currently evaluated in a clinical trial (NCT03383978). An increased ratio of anti-tumorous M1- to pro-tumorous M2-polarized macrophages within the tumor microenvironment translates into better survival in CNS lymphoma [72]. CAR-macrophages may shape the immunosuppressive tumor microenvironment by expressing pro-inflammatory cytokines and converting M2 macrophages into the pro-inflammatory M1 subpopulation [78]. Moreover, CAR NK-cells and CAR macrophages might be available as ‘off-the-shelf’ products, therefore omitting lymphapheresis. This might be particularly in CNS lymphoma patients given the often-encountered high pre-treatment burden potentially resulting impaired T-cell quality or quantity [79].

### 4.2. Combination with Other (Immunotherapeutic) Approaches

Given the peculiar role of metabolic and physical barriers for CAR T-cell efficiency in brain tumors, as well as other solid tumors outside the CNS, the combination of CAR T-cells with other therapies to ameliorate such effects might be helpful in increasing therapeutic effects. CNS lymphoma frequently shows PD-L1 expression, and PD-1-positive T-cells in the tumor microenvironment showed increased exhaustion markers compared to PD-1-negative T-cells [72]. Based on this consideration, the combination of CAR T-cells with checkpoint inhibition, approaches to reduce immunosuppressive cells in the tumor microenvironment, and therapy directed against the TAM/M receptor CSF-1R were investigated. As such, preclinical studies in brain tumor models (other than CNS lymphoma) showed increased anti-tumor effects when CAR T-cells were combined with immune checkpoint-blocking antibodies directed against the PD1-PDL1-axis or when CARs were additionally equipped with a PD1-directed domain [31]. Accordingly, clinical studies of such an approach are currently ongoing, including a trial for CD19-PD1-directed CAR T-cells in lymphoma patients without excluding patients with CNS disease (NCT04163302). In another phase I clinical trial by Chong et al., patients with refractory diffuse large B cell lymphoma received cyclophosphamide chemotherapy followed by autologous CD19 CAR T-cell administration. A patient was from this trial was separately reported: At the time of progression, the patient’s tumor cells expressed high levels of PD-L1. Therefore, on day 26 after T-cell infusion, pembrolizumab was administered every 3 weeks (2 mg/kg). The investigators observed decreasing numbers of T-cells expressing PD-1, and a regression of multiple lesions by day 45 [80]. On a cautionary note, other studies (e.g., on neuroblastoma) did not observe a clinical benefit for augmenting CAR T-cells with PD-1 inhibition [81]. This approach therefore warrants careful and thorough evaluation, and the results from ongoing studies are expected to deliver evidence whether checkpoint inhibition indeed increases CAR T-cell efficiency in CNS lymphoma.

Colony-stimulating factor 1 receptor (CSF-1R) controls the formation, differentiation, and function of M2 macrophages and strategies to limit myeloid recruitment or reprogram the myeloid populations by CSF-1R-targeting have been proven beneficial [82,83]. In preclinical studies on CNS tumors, the blockade of CSF-1R enhanced therapeutic efficiency of immune checkpoint blockade by reducing recruitment of bone marrow-derived macrophages [84]. One may therefore speculate that selective targeting of CSF-1R by CSF-1R-blocking agents or by CAR T-/NK-cells may synergize with CAR therapy [85,86].

Similar to CAR T-cell delivery to CNS tumor sites, the ideal mode of administration of these additional combinatorial therapies is still an area of uncertainty. Different mechanisms such as direct injection via viral vectors or polymer systems using reservoir systems but also infusion via convection-enhanced delivery are currently being investigated [87,88].

## 5. Conclusions

In conclusion, CAR T-cells appear as a promising therapeutic approach in CNS lymphoma. Preclinical data seem promising, but there is a lack of in vivo studies in immunocompetent animals. Moreover, adverse effects of CAR T-cell therapy are not adequately depictured in animal models and need peculiar attention when translation this therapy into clinical studies [45]. Based on the limited clinical data available for CNS lymphoma, anti-tumor effects and an acceptable side effect profile might be assumed. However, whether anti-tumor effects are durable is questionable. In addition, it is unclear whether the existence of beneficial anti-tumor effects is true for both patients with primary and secondary CNS lymphoma given unique differences in pathogenesis and clinical characteristics between the two diseases [89]. Dedicated clinical trials on CAR T-cells for PCNSL patients are therefore urgently warranted. Immune-escaping properties of CNS lymphomas might be among the most relevant factors limiting CAR T-cell efficacy in the CNS. Modifications of CAR design and the combination of CAR T-cell therapy with other therapeutic approaches may pave the way to clinical relevance of such therapy in CNS lymphoma.

## Figures and Tables

**Table 1:** Published studies on CAR T-cells for treatment of primary and secondary CNS lymphoma.

	Study Design	Study Population	Route of Delivery	Antigens	Toxicities	Outcome	NCT/ ChiCTR
Abramson et al. [19]	Case report on a patient enrolled in a phase 1 clinical trial	Secondary CNS lymphoma (*n* = 1): Diffuse large B-cell lymphoma	Intravenously	Lisocabtagene maraleucel (formerly JCAR017): ^CD19^CAR T-cells	None	CR after 1 months	NCT02631044
Frigault et al. [20]	Retrospective cohort study	Secondary CNS lymphoma (*n* = 8): Diffuse large B-cell lymphoma (*n* = 5) High-grade B-cell lymphoma (*n* = 2) Primary mediastinal B-cell lymphoma (*n* = 1)	Intravenously	Tisagenlecleucel: ^CD19^CAR T-cells	Grade 1 CRS (*n* = 7) No NT No tocilizumab or steroid treatment needed	PD (*n* = 4) with † on day 3 and 25 (*n* = 2) PR (*n* = 2) with ongoing control on day 90 (*n* = 1) and 180 (*n* = 1) CR (*n* = 2) with ongoing control on day 90 (*n* = 1) 180 (*n* = 1)	NCT04134117
Siddiqi et al. [21]	Preliminary data from an ongoing phase 1 clinical trial	Primary CNS lymphoma (*n* = 3) Secondary CNS lymphoma (*n* = 4)	Intravenously (*n* = 7) Intraventricular, under evaluation	^CD19^CAR T-cells modified to express a truncated eGFR	Grade 1–2 NT and CRS, treated with steroids (*n* = 2) or tocilizumab (*n* = 3)	CR (*n* = 1) PR (*n* = 3)	NCT02153580
Li et al. [22]	Phase 1 clinical trial	Primary CNS lymphoma (*n* = 1) Secondary CNS lymphoma (*n* = 4)	Intravenously	Combination of: ^CD19^CAR T-cells ^CD22^CAR T-cells	Grade 1 (*n* = 4) and 2 (*n* = 1) CRS Grade 1 (n = 1) and 4 (*n* = 1) NT, treated with steroids, plasmapheresis, tocilizumab	60-days assessment: CR (*n* = 1) PR (*n* = 4)	ChiCTR-OPN-16008526

Study design, study population, route of CAR T-cell delivery, antigens, toxicities, patient outcome, and NCT/ChiCTR are indicated. Maximum CRS and NT were graded according to ASTCT [23]. Abbreviations: ASTCT—American Society for Transplantation and Cellular Therapy. ChiCTR—Chinese clinical trial register. CNS—central nervous system. CR—complete response. CRS—cytokine release syndrome. NCT—national clinical trial identifier. NT—neurotoxicity. PD—progressive disease. PR—partial response.

**Table 2:** Current clinical trials to primary and secondary CNS lymphoma.

Sponsor	Study Chair	Study Design	Population	Conditions	Interventions	Route of Application	NCT
University College London	Claire Roddie	Phase I clinical trial	Adults (>16 years)	Refractory/relapsed primary CNS lymphoma	Anti-CD19 CAR T-cells after lymphodepletion and pembrolizumab	Intravenously Intraventricularly via Ommaya reservoir	NCT04443829
Massachusetts General Hospital	Matthew J. Frigault	Phase I clinical trial	Adults (>18 years)	Refractory/relapsed primary CNS lymphoma	Tisagenlecleucel (anti-CD19 CAR T-cells after lymphodepletion)	Intravenously	NCT04134117
Dana-Farber Cancer Institute	Caron A. Jacobson	Phase I clinical trial	Adults (>18 years)	Refractory/relapsed central nervous system (CNS) lymphoma Systemic lymphoma with concurrent CNS lymphoma	Axicabtagene ciloleucel (anti-CD19 CAR T-cells after lymphodepletion)	Intravenously	NCT04608487
Memorial Sloan Kettering Cancer Center	Jae Park	Phase I dose-escalation trial	Adults (>18 years)	Refractory/relapsed central nervous system (CNS) lymphoma Systemic lymphoma with concurrent CNS lymphoma	Anti-CD19 19(T2)28z1XX CAR T-cells	Intravenously	NCT04464200
Celgene	Claudia Schusterbauer	Phase II clinical trial	Adults (>18 years)	Refractory/relapsed central nervous system (CNS) lymphoma Systemic lymphoma with concurrent CNS lymphoma	Lisocabtagene maraleucel (anti-CD19 CAR T-cells after lymphodepletion)	Intravenously	NCT03484702
Zhejiang University	He Huang	Early phase I clinical trial	Children (>3 years) Adults (18–75 years)	Acute lymphoblastic leukemia with CNS involvement Non-Hodgkin’s lymphoma with CNS involvement	Anti-CD19 CAR T-cells after lymphodepletion	Intraventricularly	NCT04532203

Sponsor, study chair, study design, study population, conditions, interventions, route of application, and NCT are indicated. Abbreviations: CNS—central nervous system; CAR—chimeric antigen receptor; NCT—national clinical trial identifier.

## Data Availability

No new data were created or analyzed in this study. Data sharing is not applicable to this article.

## Supplementary Materials

The following are available online at https://www.mdpi.com/article/10.3390/cancers13102503/s1, Figure S1: Diagnostic and therapeutic algorithm for suspected PCNSL.

## References

[1] Karschnia P., Batchelor T.T., Jordan J.T., Shaw B., Winter S.F., Barbiero F.J., Kaulen L.D., Thon N., Tonn J.-C., Huttner A.J. (2020). Primary Dural Lymphomas: Clinical Presentation, Management, and Outcome. Cancer.

[2] Ostrom Q.T., Gittleman H., Truitt G., Boscia A., Kruchko C., Barnholtz-Sloan J.S. (2018). CBTRUS Statistical Report: Primary Brain and Other Central Nervous System Tumors Diagnosed in the United States in 2011–2015. Neuro Oncol..

[3] Han C.H., Batchelor T.T. (2017). Diagnosis and Management of Primary Central Nervous System Lymphoma. Cancer.

[4] Ferreri A.J.M. (2011). How I Treat Primary CNS Lymphoma. Blood.

[5] Glass J., Gruber M.L., Cher L., Hochberg F.H. (1994). Preirradiation Methotrexate Chemotherapy of Primary Central Nervous System Lymphoma: Long-Term Outcome. J. Neurosurg..

[6] Houillier C., Soussain C., Ghesquières H., Soubeyran P., Chinot O., Taillandier L., Lamy T., Choquet S., Ahle G., Damaj G. (2020). Management and Outcome of Primary CNS Lymphoma in the Modern Era: An LOC Network Study. Neurology.

[7] Karschnia P., Parsons M.W., Dietrich J. (2019). Pharmacologic Management of Cognitive Impairment Induced by Cancer Therapy. Lancet Oncol..

[8] El-Galaly T.C., Cheah C.Y., Bendtsen M.D., Nowakowski G.S., Kansara R., Savage K.J., Connors J.M., Sehn L.H., Goldschmidt N., Shaulov A. (2018). Treatment Strategies, Outcomes and Prognostic Factors in 291 Patients with Secondary CNS Involvement by Diffuse Large B-Cell Lymphoma. Eur. J. Cancer.

[9] June C.H., Sadelain M. (2018). Chimeric Antigen Receptor Therapy. N. Engl. J. Med..

[10] Savoldo B., Ramos C.A., Liu E., Mims M.P., Keating M.J., Carrum G., Kamble R.T., Bollard C.M., Gee A.P., Mei Z. (2011). CD28 Costimulation Improves Expansion and Persistence of Chimeric Antigen Receptor–Modified T Cells in Lymphoma Patients. J. Clin. Investig..

[11] Schuster S.J., Bishop M.R., Tam C.S., Waller E.K., Borchmann P., McGuirk J.P., Jäger U., Jaglowski S., Andreadis C., Westin J.R. (2019). Tisagenlecleucel in Adult Relapsed or Refractory Diffuse Large B-Cell Lymphoma. N. Engl. J. Med..

[12] Maude S.L., Laetsch T.W., Buechner J., Rives S., Boyer M., Bittencourt H., Bader P., Verneris M.R., Stefanski H.E., Myers G.D. (2018). Tisagenlecleucel in Children and Young Adults with B-Cell Lymphoblastic Leukemia. N. Engl. J. Med..

[13] Wang M., Munoz J., Goy A., Locke F.L., Jacobson C.A., Hill B.T., Timmerman J.M., Holmes H., Jaglowski S., Flinn I.W. (2020). KTE-X19 CAR T-Cell Therapy in Relapsed or Refractory Mantle-Cell Lymphoma. N. Engl. J. Med..

[14] Deckert M., Montesinos-Rongen M., Brunn A., Siebert R. (2014). Systems Biology of Primary CNS Lymphoma: From Genetic Aberrations to Modeling in Mice. Acta Neuropathol..

[15] Giannini C., Dogan A., Salomão D.R. (2014). CNS Lymphoma: A Practical Diagnostic Approach. J. Neuropathol. Exp. Neurol..

[16] Mulazzani M., Fräßle S.P., von Mücke-Heim I., Langer S., Zhou X., Ishikawa-Ankerhold H., Leube J., Zhang W., Dötsch S., Svec M. (2019). Long-Term in Vivo Microscopy of CAR T Cell Dynamics during Eradication of CNS Lymphoma in Mice. Proc. Natl. Acad. Sci. USA.

[17] Hu S.-I., Ko M.-C., Dai Y.-H., Lin H.-A., Chen L.-C., Huang K.-Y., Pang T.-L., Kuo C.-Y., Lin H.-C. (2020). Pre-Clinical Assessment of Chimeric Antigen Receptor t Cell Therapy Targeting CD19+ B Cell Malignancy. Ann. Transl. Med..

[18] Wang X., Huynh C., Urak R., Weng L., Walter M., Lim L., Vyas V., Chang W.-C., Aguilar B., Brito A. (2021). The Cerebroventricular Environment Modifies CAR T Cells for Potent Activity against Both Central Nervous System and Systemic Lymphoma. Cancer Immunol. Res..

[19] Abramson J.S., McGree B., Noyes S., Plummer S., Wong C., Chen Y.-B., Palmer E., Albertson T., Ferry J.A., Arrillaga-Romany I.C. (2017). Anti-CD19 CAR T Cells in CNS Diffuse Large-B-Cell Lymphoma. N. Engl. J. Med..

[20] Frigault M.J., Dietrich J., Martinez-Lage M., Leick M., Choi B.D., DeFilipp Z., Chen Y.-B., Abramson J., Crombie J., Armand P. (2019). Tisagenlecleucel CAR T-Cell Therapy in Secondary CNS Lymphoma. Blood.

[21] Siddiqi T., Wang X., Palmer J., Popplewell L.L., Nikolaenko L., Herrera A.F., Budde L.E., Lim L., Vyas V., Brown C.E. (2019). CD19-Targeting CAR-T Cell Therapy in CNS Lymphoma. Blood.

[22] Li T., Zhao L., Zhang Y., Xiao Y., Wang D., Huang L., Ma L., Chen L., Liu S., Long X. (2020). CAR T-Cell Therapy Is Effective but Not Long-Lasting in B-Cell Lymphoma of the Brain. Front. Oncol..

[23] Lee D.W., Santomasso B.D., Locke F.L., Ghobadi A., Turtle C.J., Brudno J.N., Maus M.V., Park J.H., Mead E., Pavletic S. (2019). ASTCT Consensus Grading for Cytokine Release Syndrome and Neurologic Toxicity Associated with Immune Effector Cells. Biol. Blood Marrow Transplant..

[24] Shah N.N., Highfill S.L., Shalabi H., Yates B., Jin J., Wolters P.L., Ombrello A., Steinberg S.M., Martin S., Delbrook C. (2020). CD4/CD8 T-Cell Selection Affects Chimeric Antigen Receptor (CAR) T-Cell Potency and Toxicity: Updated Results From a Phase I Anti-CD22 CAR T-Cell Trial. J. Clin. Oncol..

[25] Sampson J.H., Gunn M.D., Fecci P.E., Ashley D.M. (2020). Brain Immunology and Immunotherapy in Brain Tumours. Nat. Rev. Cancer.

[26] Hambardzumyan D., Gutmann D.H., Kettenmann H. (2016). The Role of Microglia and Macrophages in Glioma Maintenance and Progression. Nat. Neurosci..

[27] Murray P.J., Allen J.E., Biswas S.K., Fisher E.A., Gilroy D.W., Goerdt S., Gordon S., Hamilton J.A., Ivashkiv L.B., Lawrence T. (2014). Macrophage Activation and Polarization: Nomenclature and Experimental Guidelines. Immunity.

[28] Nam S.J., Kim S., Kwon D., Kim H., Kim S., Lee E., Kim T.M., Heo D.S., Park S.H., Lim M.S. (2018). Prognostic Implications of Tumor-Infiltrating Macrophages, M2 Macrophages, Regulatory T-Cells, and Indoleamine 2,3-Dioxygenase-Positive Cells in Primary Diffuse Large B-Cell Lymphoma of the Central Nervous System. Oncoimmunology.

[29] Korfel A., Schlegel U. (2013). Diagnosis and Treatment of Primary CNS Lymphoma. Nat. Rev. Neurol..

[30] Chmielewski M., Kopecky C., Hombach A.A., Abken H. (2011). IL-12 Release by Engineered T Cells Expressing Chimeric Antigen Receptors Can Effectively Muster an Antigen-Independent Macrophage Response on Tumor Cells That Have Shut down Tumor Antigen Expression. Cancer Res..

[31] Shen S.H., Woroniecka K., Barbour A.B., Fecci P.E., Sanchez-Perez L., Sampson J.H. (2020). CAR T Cells and Checkpoint Inhibition for the Treatment of Glioblastoma. Exp. Opin. Biol. Ther..

[32] Santomasso B.D., Park J.H., Salloum D., Riviere I., Flynn J., Mead E., Halton E., Wang X., Senechal B., Purdon T. (2018). Clinical and Biological Correlates of Neurotoxicity Associated with CAR T-Cell Therapy in Patients with B-Cell Acute Lymphoblastic Leukemia. Cancer Discov..

[33] Keu K.V., Witney T.H., Yaghoubi S., Rosenberg J., Kurien A., Magnusson R., Williams J., Habte F., Wagner J.R., Forman S. (2017). Reporter Gene Imaging of Targeted T Cell Immunotherapy in Recurrent Glioma. Sci. Transl. Med..

[34] Sampson J.H., Choi B.D., Sanchez-Perez L., Suryadevara C.M., Snyder D.J., Flores C.T., Schmittling R.J., Nair S.K., Reap E.A., Norberg P.K. (2014). EGFRvIII MCAR-Modified T-Cell Therapy Cures Mice with Established Intracerebral Glioma and Generates Host Immunity against Tumor-Antigen Loss. Clin. Cancer Res..

[35] Brown C.E., Alizadeh D., Starr R., Weng L., Wagner J.R., Naranjo A., Ostberg J.R., Blanchard M.S., Kilpatrick J., Simpson J. (2016). Regression of Glioblastoma after Chimeric Antigen Receptor T-Cell Therapy. N. Engl. J. Med..

[36] Turtle C.J., Hanafi L.-A., Berger C., Gooley T.A., Cherian S., Hudecek M., Sommermeyer D., Melville K., Pender B., Budiarto T.M. (2016). CD19 CAR-T Cells of Defined CD4+:CD8+ Composition in Adult B Cell ALL Patients. J. Clin. Investig..

[37] Zhang Z., Chen X., Tian Y., Li F., Zhao X., Liu J., Yao C., Zhang Y. (2020). Point Mutation in CD19 Facilitates Immune Escape of B Cell Lymphoma from CAR-T Cell Therapy. J. Immunother. Cancer.

[38] Nayyar N., White M.D., Gill C.M., Lastrapes M., Bertalan M., Kaplan A., D’Andrea M.R., Bihun I., Kaneb A., Dietrich J. (2019). MYD88 L265P Mutation and CDKN2A Loss Are Early Mutational Events in Primary Central Nervous System Diffuse Large B-Cell Lymphomas. Blood Adv..

[39] Neelapu S.S., Tummala S., Kebriaei P., Wierda W., Gutierrez C., Locke F.L., Komanduri K.V., Lin Y., Jain N., Daver N. (2018). Chimeric Antigen Receptor T-Cell Therapy—Assessment and Management of Toxicities. Nat. Rev. Clin. Oncol..

[40] Wudhikarn K., Pennisi M., Garcia-Recio M., Flynn J.R., Afuye A., Silverberg M.L., Maloy M.A., Devlin S.M., Batlevi C.L., Shah G.L. (2020). DLBCL Patients Treated with CD19 CAR T Cells Experience a High Burden of Organ Toxicities but Low Nonrelapse Mortality. Blood Adv..

[41] Fajgenbaum D.C., June C.H. (2020). Cytokine Storm. N. Engl. J. Med..

[42] Sokolov E., Karschnia P., Benjamin R., Hadden R.D.M., Elwes R.C.D., Drummond L., Amin D., Paiva V., Pennisi A., Herlopian A. (2020). Language Dysfunction-Associated EEG Findings in Patients with CAR-T Related Neurotoxicity. BMJ Neurol. Open.

[43] Karschnia P., Strübing F., Teske N., Blumenberg V., Bücklein V.L., Schmidt C., Schöberl F., Dimitriadis K., Forbrig R., Stemmler H.-J. (2021). Clinicopathologic Findings in Fatal Neurotoxicity After Adoptive Immunotherapy With CD19-Directed CAR T-Cells. Hemasphere.

[44] Norelli M., Camisa B., Barbiera G., Falcone L., Purevdorj A., Genua M., Sanvito F., Ponzoni M., Doglioni C., Cristofori P. (2018). Monocyte-Derived IL-1 and IL-6 Are Differentially Required for Cytokine-Release Syndrome and Neurotoxicity Due to CAR T Cells. Nat. Med..

[45] Parker K.R., Migliorini D., Perkey E., Yost K.E., Bhaduri A., Bagga P., Haris M., Wilson N.E., Liu F., Gabunia K. (2020). Single-Cell Analyses Identify Brain Mural Cells Expressing CD19 as Potential Off-Tumor Targets for CAR-T Immunotherapies. Cell.

[46] Karschnia P., Jordan J.T., Forst D.A., Arrillaga-Romany I.C., Batchelor T.T., Baehring J.M., Clement N.F., Gonzalez Castro L.N., Herlopian A., Maus M.V. (2019). Clinical Presentation, Management, and Biomarkers of Neurotoxicity after Adoptive Immunotherapy with CAR T Cells. Blood.

[47] Xiao X., He X., Li Q., Zhang H., Meng J., Jiang Y., Deng Q., Zhao M. (2019). Plasma Exchange Can Be an Alternative Therapeutic Modality for Severe Cytokine Release Syndrome after Chimeric Antigen Receptor-T Cell Infusion: A Case Report. Clin. Cancer Res..

[48] Strati P., Ahmed S., Kebriaei P., Nastoupil L.J., Claussen C.M., Watson G., Horowitz S.B., Brown A.R.T., Do B., Rodriguez M.A. (2020). Clinical Efficacy of Anakinra to Mitigate CAR T-Cell Therapy-Associated Toxicity in Large B-Cell Lymphoma. Blood Adv..

[49] Hill J.A., Giralt S., Torgerson T.R., Lazarus H.M. (2019). CAR-T—and a Side Order of IgG, to Go?—Immunoglobulin Replacement in Patients Receiving CAR-T Cell Therapy. Blood Rev..

[50] Goff S.L., Morgan R.A., Yang J.C., Sherry R.M., Robbins P.F., Restifo N.P., Feldman S.A., Lu Y.-C., Lu L., Zheng Z. (2019). Pilot Trial of Adoptive Transfer of Chimeric Antigen Receptor-Transduced T Cells Targeting EGFRvIII in Patients With Glioblastoma. J. Immunother..

[51] Ruark J., Mullane E., Cleary N., Cordeiro A., Bezerra E.D., Wu V., Voutsinas J., Shaw B.E., Flynn K.E., Lee S.J. (2020). Patient-Reported Neuropsychiatric Outcomes of Long-Term Survivors after Chimeric Antigen Receptor T Cell Therapy. Biol. Blood Marrow Transplant..

[52] Restifo N.P., Dudley M.E., Rosenberg S.A. (2012). Adoptive Immunotherapy for Cancer: Harnessing the T Cell Response. Nat. Rev. Immunol..

[53] Jain T., Knezevic A., Pennisi M., Chen Y., Ruiz J.D., Purdon T.J., Devlin S.M., Smith M., Shah G.L., Halton E. (2020). Hematopoietic Recovery in Patients Receiving Chimeric Antigen Receptor T-Cell Therapy for Hematologic Malignancies. Blood Adv..

[54] Rejeski K., Kunz W.G., Rudelius M., Bücklein V., Blumenberg V., Schmidt C., Karschnia P., Schöberl F., Dimitriadis K., von Baumgarten L. (2021). Severe Candida Glabrata Pancolitis and Fatal Aspergillus Fumigatus Pulmonary Infection in the Setting of Bone Marrow Aplasia after CD19-Directed CAR T-Cell Therapy—A Case Report. BMC Infect. Dis..

[55] Sterner R.M., Sakemura R., Cox M.J., Yang N., Khadka R.H., Forsman C.L., Hansen M.J., Jin F., Ayasoufi K., Hefazi M. (2019). GM-CSF Inhibition Reduces Cytokine Release Syndrome and Neuroinflammation but Enhances CAR-T Cell Function in Xenografts. Blood.

[56] Ferreri A.J.M., Illerhaus G. (2016). The Role of Autologous Stem Cell Transplantation in Primary Central Nervous System Lymphoma. Blood.

[57] Illerhaus G., Kasenda B., Ihorst G., Egerer G., Lamprecht M., Keller U., Wolf H.-H., Hirt C., Stilgenbauer S., Binder M. (2016). High-Dose Chemotherapy with Autologous Haemopoietic Stem Cell Transplantation for Newly Diagnosed Primary CNS Lymphoma: A Prospective, Single-Arm, Phase 2 Trial. Lancet Haematol..

[58] Kasenda B., Ihorst G., Schroers R., Korfel A., Schmidt-Wolf I., Egerer G., von Baumgarten L., Röth A., Bloehdorn J., Möhle R. (2017). High-Dose Chemotherapy with Autologous Haematopoietic Stem Cell Support for Relapsed or Refractory Primary CNS Lymphoma: A Prospective Multicentre Trial by the German Cooperative PCNSL Study Group. Leukemia.

[59] Korell F., Laier S., Sauer S., Veelken K., Hennemann H., Schubert M.-L., Sauer T., Pavel P., Mueller-Tidow C., Dreger P. (2020). Current Challenges in Providing Good Leukapheresis Products for Manufacturing of CAR-T Cells for Patients with Relapsed/Refractory NHL or ALL. Cells.

[60] Kotani H., Li G., Yao J., Mesa T.E., Chen J., Boucher J.C., Yoder S.J., Zhou J., Davila M.L. (2018). Aged CAR T Cells Exhibit Enhanced Cytotoxicity and Effector Function but Shorter Persistence and Less Memory-like Phenotypes. Blood.

[61] Kaulen L.D., Karschnia P., Dietrich J., Baehring J.M. (2020). Autoimmune Disease-Related Primary CNS Lymphoma: Systematic Review and Meta-Analysis. J. Neurooncol..

[62] Kaulen L.D., Erson-Omay E.Z., Henegariu O., Karschnia P., Huttner A., Günel M., Baehring J.M. (2021). Exome Sequencing Identifies SLIT2 Variants in Primary CNS Lymphoma. Br. J. Haematol..

[63] Kaulen L.D., Galluzzo D., Hui P., Barbiero F., Karschnia P., Huttner A., Fulbright R., Baehring J.M. (2019). Prognostic Markers for Immunodeficiency-Associated Primary Central Nervous System Lymphoma. J. Neurooncol..

[64] Jhaveri K.S., Schlam I., Holtzman N.G., Peravali M., Richardson P.K., Dahiya S., Malkovska V., Rapoport A.P. (2020). Safety and Efficacy of CAR T Cells in a Patient with Lymphoma and a Coexisting Autoimmune Neuropathy. Blood Adv..

[65] Kershaw M.H., Westwood J.A., Parker L.L., Wang G., Eshhar Z., Mavroukakis S.A., White D.E., Wunderlich J.R., Canevari S., Rogers-Freezer L. (2006). A Phase I Study on Adoptive Immunotherapy Using Gene-Modified T Cells for Ovarian Cancer. Clin. Cancer Res..

[66] Brocker T., Karjalainen K. (1995). Signals through T Cell Receptor-Zeta Chain Alone Are Insufficient to Prime Resting T Lymphocytes. J. Exp. Med..

[67] Maher J., Brentjens R.J., Gunset G., Rivière I., Sadelain M. (2002). Human T-Lymphocyte Cytotoxicity and Proliferation Directed by a Single Chimeric TCRzeta /CD28 Receptor. Nat. Biotechnol..

[68] Imai C., Mihara K., Andreansky M., Nicholson I.C., Pui C.-H., Geiger T.L., Campana D. (2004). Chimeric Receptors with 4-1BB Signaling Capacity Provoke Potent Cytotoxicity against Acute Lymphoblastic Leukemia. Leukemia.

[69] Till B.G., Jensen M.C., Wang J., Qian X., Gopal A.K., Maloney D.G., Lindgren C.G., Lin Y., Pagel J.M., Budde L.E. (2012). CD20-Specific Adoptive Immunotherapy for Lymphoma Using a Chimeric Antigen Receptor with Both CD28 and 4-1BB Domains: Pilot Clinical Trial Results. Blood.

[70] Zhong X.-S., Matsushita M., Plotkin J., Riviere I., Sadelain M. (2010). Chimeric Antigen Receptors Combining 4-1BB and CD28 Signaling Domains Augment PI3kinase/AKT/Bcl-XL Activation and CD8+ T Cell-Mediated Tumor Eradication. Mol. Ther..

[71] Wang J., Jensen M., Lin Y., Sui X., Chen E., Lindgren C.G., Till B., Raubitschek A., Forman S.J., Qian X. (2007). Optimizing Adoptive Polyclonal T Cell Immunotherapy of Lymphomas, Using a Chimeric T Cell Receptor Possessing CD28 and CD137 Costimulatory Domains. Hum. Gene Ther..

[72] Marcelis L., Antoranz A., Delsupehe A.-M., Biesemans P., Ferreiro J.F., Debackere K., Vandenberghe P., Verhoef G., Gheysens O., Cattoretti G. (2020). In-Depth Characterization of the Tumor Microenvironment in Central Nervous System Lymphoma Reveals Implications for Immune-Checkpoint Therapy. Cancer Immunol. Immunother..

[73] Chmielewski M., Abken H. (2015). TRUCKs: The Fourth Generation of CARs. Expert Opin. Biol. Ther..

[74] Tu S., Zhou X., Guo Z., Huang R., Yue C., He Y., Li M., Chen Y., Liu Y., Chang L.-J. (2019). CD19 and CD70 Dual-Target Chimeric Antigen Receptor T-Cell Therapy for the Treatment of Relapsed and Refractory Primary Central Nervous System Diffuse Large B-Cell Lymphoma. Front. Oncol..

[75] Oei V.Y.S., Siernicka M., Graczyk-Jarzynka A., Hoel H.J., Yang W., Palacios D., Almåsbak H., Bajor M., Clement D., Brandt L. (2018). Intrinsic Functional Potential of NK-Cell Subsets Constrains Retargeting Driven by Chimeric Antigen Receptors. Cancer Immunol. Res..

[76] Liu E., Marin D., Banerjee P., Macapinlac H.A., Thompson P., Basar R., Nassif Kerbauy L., Overman B., Thall P., Kaplan M. (2020). Use of CAR-Transduced Natural Killer Cells in CD19-Positive Lymphoid Tumors. N. Engl. J. Med..

[77] Burger M.C., Zhang C., Harter P.N., Romanski A., Strassheimer F., Senft C., Tonn T., Steinbach J.P., Wels W.S. (2019). CAR-Engineered NK Cells for the Treatment of Glioblastoma: Turning Innate Effectors Into Precision Tools for Cancer Immunotherapy. Front. Immunol..

[78] Klichinsky M., Ruella M., Shestova O., Lu X.M., Best A., Zeeman M., Schmierer M., Gabrusiewicz K., Anderson N.R., Petty N.E. (2020). Human Chimeric Antigen Receptor Macrophages for Cancer Immunotherapy. Nat. Biotechnol..

[79] Basar R., Daher M., Rezvani K. (2020). Next-Generation Cell Therapies: The Emerging Role of CAR-NK Cells. Blood Adv..

[80] Chong E.A., Melenhorst J.J., Lacey S.F., Ambrose D.E., Gonzalez V., Levine B.L., June C.H., Schuster S.J. (2017). PD-1 Blockade Modulates Chimeric Antigen Receptor (CAR)-Modified T Cells: Refueling the CAR. Blood.

[81] Heczey A., Louis C.U., Savoldo B., Dakhova O., Durett A., Grilley B., Liu H., Wu M.F., Mei Z., Gee A. (2017). CAR T Cells Administered in Combination with Lymphodepletion and PD-1 Inhibition to Patients with Neuroblastoma. Mol. Ther..

[82] Yan D., Kowal J., Akkari L., Schuhmacher A.J., Huse J.T., West B.L., Joyce J.A. (2017). Inhibition of Colony Stimulating Factor-1 Receptor Abrogates Microenvironment-Mediated Therapeutic Resistance in Gliomas. Oncogene.

[83] Poh A.R., Ernst M. (2018). Targeting Macrophages in Cancer: From Bench to Bedside. Front. Oncol..

[84] Aslan K., Turco V., Blobner J., Sonner J.K., Liuzzi A.R., Núñez N.G., De Feo D., Kickingereder P., Fischer M., Green E. (2020). Heterogeneity of Response to Immune Checkpoint Blockade in Hypermutated Experimental Gliomas. Nat. Commun..

[85] Hussain S.F., Yang D., Suki D., Aldape K., Grimm E., Heimberger A.B. (2006). The Role of Human Glioma-Infiltrating Microglia/Macrophages in Mediating Antitumor Immune Responses. Neuro Oncol..

[86] Xin H., Zhang C., Herrmann A., Du Y., Figlin R., Yu H. (2009). Sunitinib Inhibition of Stat3 Induces Renal Cell Carcinoma Tumor Cell Apoptosis and Reduces Immunosuppressive Cells. Cancer Res..

[87] Lonser R.R., Akhter A.S., Zabek M., Elder J.B., Bankiewicz K.S. (2020). Direct Convective Delivery of Adeno-Associated Virus Gene Therapy for Treatment of Neurological Disorders. J. Neurosurg..

[88] Kunigelis K.E., Vogelbaum M.A. (2021). Therapeutic Delivery to Central Nervous System. Neurosurg. Clin. N. Am..

[89] Rubenstein J.L., Gupta N.K., Mannis G.N., Lamarre A.K., Treseler P. (2013). How I Treat CNS Lymphomas. Blood.

