# Supplementary material for: CAR T-Cells for CNS Lymphoma: Driving into New Terrain?
Source: Cancers (Basel). 2021 May 20;13(10):2503. doi: 10.3390/cancers13102503 (PMC8161128; doi:10.3390/cancers13102503)
Supplement: Supplementary file 1 [file cancers-13-02503-s001.zip › cancers-1207106-supplementary.pdf]

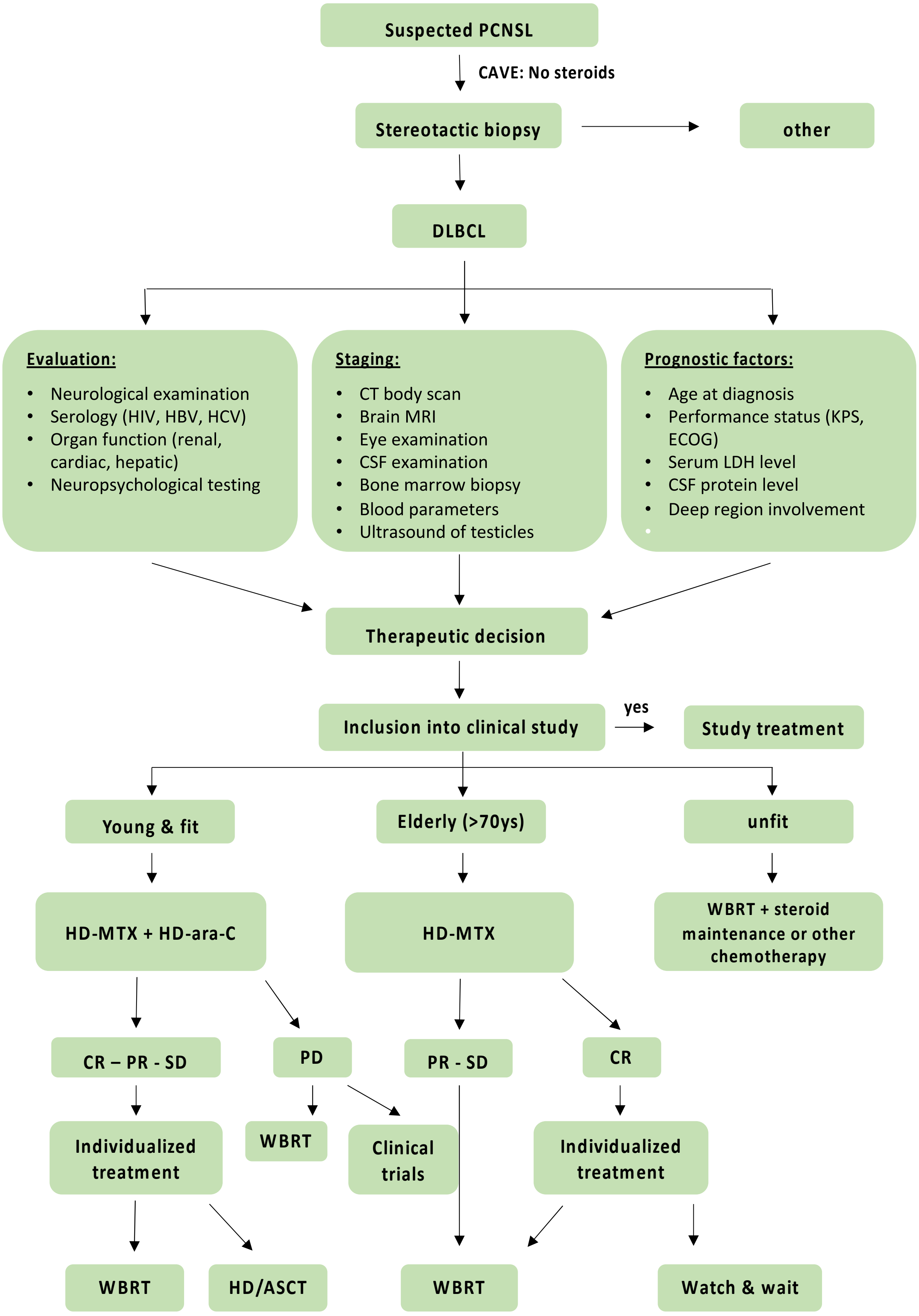

Supplementary Figure 1: Diagnostic and therapeutic algorithm for suspected PCNSL. MRI: magnet resonance imaging; HIV: human immunodeficiency virus; HBV: hepatitis B virus; HCV: hepatitis C virus; CT: computer tomography; CSF: cerebrospinal fluid; KPS: Karnofsky Performance index; LDH: Lactate dehydrogenase; HD-MTX: high-dosage Methotrexate: HD-ara-C: high-

dosage cytarabine; CR: complete remission; PR: partial remission; SD: stable disease; CR: complete remission; WBRT: whole brain radiotherapy; ASCT: autologous stem cell therapy. Modified from Ferreri et al. (2011).
